# Extracellular Vehicles of Oxygen-Depleted Mesenchymal Stromal Cells: Route to Off-Shelf Cellular Therapeutics?

**DOI:** 10.3390/cells10092199

**Published:** 2021-08-26

**Authors:** Dhir Gala, Sidhesh Mohak, Zsolt Fábián

**Affiliations:** Department of Molecular Cell Biology, School of Medicine, Faculty of Clinical and Biomedical Sciences, University of Central Lancashire, Preston PR1 1JQ, UK; dhirgala@students.aucmed.edu (D.G.); SMohak@uclan.ac.uk (S.M.)

**Keywords:** bone marrow-derived stromal cells, hypoxia, extracellular vesicles

## Abstract

Cellular therapy is a promising tool of human medicine to successfully treat complex and challenging pathologies such as cardiovascular diseases or chronic inflammatory conditions. Bone marrow-derived mesenchymal stromal cells (BMSCs) are in the limelight of these efforts, initially, trying to exploit their natural properties by direct transplantation. Extensive research on the therapeutic use of BMSCs shed light on a number of key aspects of BMSC physiology including the importance of oxygen in the control of BMSC phenotype. These efforts also led to a growing number of evidence indicating that the beneficial therapeutic effects of BMSCs can be mediated by BMSC-secreted agents. Further investigations revealed that BMSC-excreted extracellular vesicles could mediate the potentially therapeutic effects of BMSCs. Here, we review our current understanding of the relationship between low oxygen conditions and the effects of BMSC-secreted extracellular vesicles focusing on the possible medical relevance of this interplay.

## 1. Introduction

### 1.1. Bone Marrow-Derived Stromal Cells

**Figure 1:**
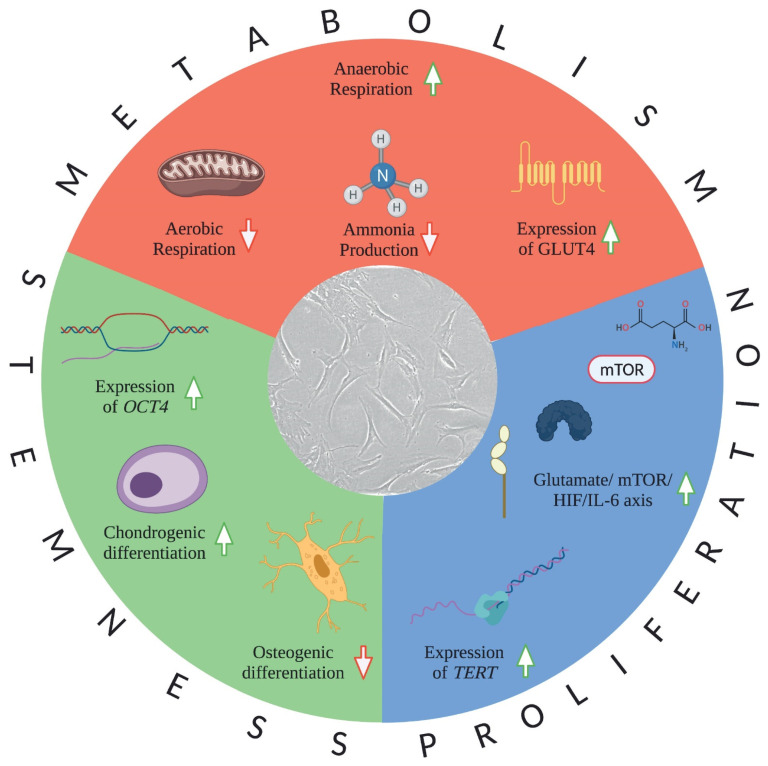
Adaptive measures of the bone marrow-derived stromal cells in hypoxic conditions. BMSC show complex adaptive measures under hypoxic conditions that affect practically all aspects of their physiology. Abbreviations used are: GLUT4: glucose transporter type 4; mTOR: mechanistic target of rapamycin; HIF: hypoxia inducible factor; IL-6: interleukin-6; TERT: telomerase reverse tran-scriptase; OCT4: octamer-binding protein 4. Micrograph of the human bone marrow-derived stomal cells are made by Zsolt Fabian. Figure was created using BioRender.com.

Adult stem cells are present in a variety of organs, where the local microenvironment has a significant impact on their capacity to differentiate [1]. One of these specialized environments is the bone marrow, which harbors both hemopoietic and non-hematopoietic stem cell species [2]. The latter ones, termed bone marrow-derived mesenchymal stromal cells (BMSCs), were first identified by Friedenstein in 1976 [2]. In vitro, the plastic adherent BMSCs show fibroblastic morphology (Figure 1) and express CD105, CD73 and CD90 [3]. BMSCs are multipotent cells with the ability to develop into a variety of cells with mesenchymal origin including adipocytes, chondro- or osteoblasts [4]. Since BMSCs are negative for CD45, CD34, CD14 or CD11b, CD79α, CD19 and HLA-DR, they show no alloreactivity in lymphocyte proliferative assays, making them ideal for cellular therapy applications [5,6]. Human BMSCs (hBMSCs) develop into pericytes, myofibroblasts, osteocytes and other mature cells that contribute to the establishment of the bone marrow microenvironment playing a role in controlling hemopoiesis via maintaining the hemopoietic microenvironment [2]. Indeed, transplantation of hBMSCs into murine bone marrow results in the reconstruction of the human bone marrow microenvironment and production of primitive human hemopoietic cells indicating the pivotal role of BMSCs in the establishment and maintenance of the bone marrow milieu [7,8]. Since one of the characteristics of this niche is low oxygenation and BMSCs reside in areas that are minimally vascularized, it is widely accepted that hypoxia is an important factor of the BMSC physiology [2].

### 1.2. Hypoxia

**Figure 2:**
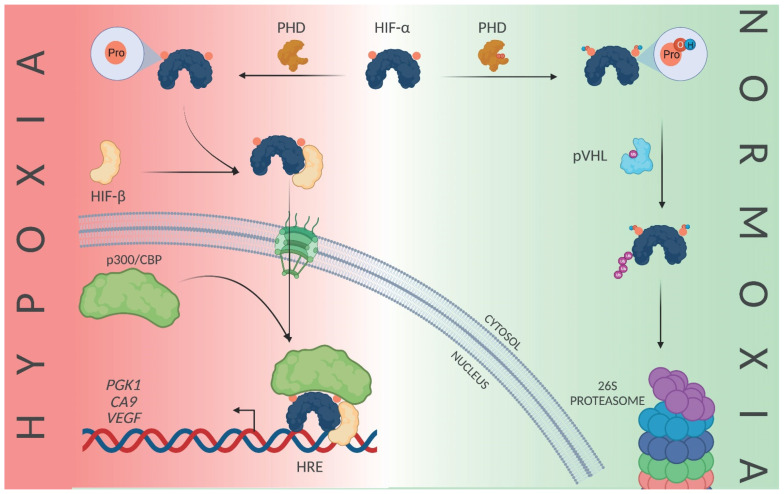
Schematic overview of the hypoxia signaling. In the absence of oxygen, prolyl hydroxylases (PHD) fail to hydroxylate the alpha subunits of the hypoxia-inducible factors (HIF), which leads to HIF dimerization in the cytosol, thus resulting in the formation of the transcriptionally active HIF and the adaptive gene expression changes. In the presence of oxygen, HIF undergoes prolyl-hydroxylation by PHD, which is followed by ubiquitination by von Hippel–Lindau protein, ultimately leading to its proteosomal degradation. Abbreviations used are: PHD: prolyl hydroxylases; HIF: hypoxia-inducible factor; pVHL: von Hippel–Lindau protein; HRE: hypoxia response element. Figure was created using BioRender.com.

Since oxygen plays a critical role in cellular metabolism, metazoan cells developed a complex molecular system to respond to oxygen deprivation. The master regulators of the adaptive measures are the hypoxia-inducible factors (HIF), which were first identified as nuclear factors interacting with the 3′ enhancer sequence of the erythropoietin gene (*EPO*) in response to hypoxia [9]. HIFs are made up of alpha (HIFα) and beta (HIFβ) subunits of which the former one is under the control of oxygen levels [10] (Figure 2). To date, three paralogues of the alpha (HIF-1α, HIF-2α and HIF-3α) and three isoforms of the beta (HIF-1β, HIF-2β and HIF-3β) subunits have been identified. The best studied heterodimer is the HIF-1 that seems to be primarily responsible for the upregulation of glycolytic genes and angiogenesis [11,12]. In contrast, the transactivation capacity of the HIF-2 heterodimer seems to be more biased toward genes that control cellular physiologic processes such as the cell cycle or stem cell pluripotency [13,14]. The HIF-3 isoform, however, both structurally and functionally, more substantially differs from HIF-1 and -2. Due to the lack of the *C*-terminal transactivation domain, splicing variants of HIF-3 have been found to counteract the hypoxia-responses of HIF-1 and -2 in a tissue-specific manner suggesting its role in the control of HIF-1 and-2 activation [15,16].

In the presence of oxygen, HIFα is hydroxylated on conserved proline residues by prolyl hydroxylase domain proteins (PHDs), the major intracellular oxygen sensors [17]. Hydroxylated HIFα undergoes a conformational change, revealing a binding site for the von Hippel–Lindau (pVHL) ubiquitin ligase that mediates polyubiquitylation and, consequently, the proteasomal degradation of the α subunit [17,18]. In hypoxia, HIF’s destabilization fails, resulting in dimerization with the β subunit and the HIF-mediated upregulation of a wide range of hypoxia-adaptive genes (recently reviewed in [19]). These include ones encoding for glycolytic enzymes such as the phosphoglycerate kinase-1 (*PGK1*) and lactate dehydrogenase A (*LDHA*), glucose and lactic acid transporters such as the *GLUT4* and the monocarboxylate transporter MCT4 (*SLC16A3*), respectively, or intracellular pH regulators such as the Na^+^/H^+^ exchanger-encoding *SLC9A1* or the carbonic anhydrase IX-encoding *CA9*, ultimately shifting the metabolism from aerobic to anaerobic [20,21,22]. In addition, HIF signaling contributes to the restoration of the oxygen supply at the supracellular level as well via induction of angiogenetic agents such as the vascular endothelial growth factor (*VEGF*) [23]. Although oxygen deprivation is traditionally linked to pathologic states, hypoxia is necessary for organogenesis and maintenance of tissue homeostasis as well [24,25,26]. Indeed, embryonic stem cells require a hypoxic environment to preserve pluripotency, implying that oxygen plays a key role in inherent stem cell traits [27]. Since HIF signaling is active in hypoxia-exposed hBMSCs, it is widely believed that the HIF-mediated machinery is involved in the development of their hypoxic phenotype (Figure 2) [28].

### 1.3. The Effects of Hypoxia on BMSCs

BMSCs cultured under low oxygen tensions consume half the amount of oxygen compared to cells grown under atmospheric oxygen conditions, suggesting the dominance of anaerobic metabolism in hypoxic BMSCs [29]. In support of this, hypoxic BMSCs show increased glucose transporter expression, elevated glucose uptake and decreased conversion of glucose carbons into the tricarboxylic acid cycle (TCA) [30,31,32]. Indeed, under normal oxygen tension, glutamate is converted to α-ketoglutarate which feeds into the TCA cycle by the glutamate dehydrogenase producing ammonia [33]. Under hypoxic conditions, however, reduced ammonia production was observed in hypoxic BMSC cultures, indicating repressed incorporation of glutamate into the TCA cycle [30]. Interestingly, due to the toxic nature of ammonia in cell cultures, its low-rate production is believed to be one of the factors that determines the proliferative phenotype of oxygen-depleted BMSCs [34,35]. Indeed, BMSCs, have faster proliferation rates at 2% than at atmospheric oxygen levels [36]. Considering that proliferation generally inhibits differentiation, one could speculate that hypoxia affects the ability of BMSCs to differentiate [37]. To support this idea, BMSCs show significantly greater expression of stem cell factors under hypoxic conditions than that of the ones cultured at ambient oxygen levels [38].

In accordance, data indicate that the hypoxic nature of bone marrow supports the stem cell phenotype of BMSCs, since the stemness marker octamer-binding protein 4 (*OCT4*) and the telomerase reverse transcriptase (*TERT*) are both induced in oxygen-depleted BMSCs [39]. In support of this idea, BMSCs cultured at low oxygen levels fail to differentiate into osteogenic lineage, show decreased calcification and repression of genes known to be involved in the osteogenic differentiation including the alkaline phosphatase (*ALPL*), RUNX family transcription factor 2 (*RUNX2*), osteocalcin (*BGLAP*) and type I collagen (*COLI*) [40]. In accordance, the osteogenesis regulator RUNX2 increases the expression of VEGF by directly inducing *HIF-1α* in various models [41,42]. These data suggest that RUNX2 acts upstream of HIF in the osteogenic context and activate the HIF pathway to provide the oxygen supply for osteogenic differentiation. According to this concept, the HIF-mediated induction of SOX9, the transcription factor that governs chondrogenesis, could reflect that chondrogenic differentiation is supported by the hypoxic milieu [43,44]. Indeed, BMSCs exposed to hypoxia show better chondrogenic potential in vitro [45]. Similarly, the induction of adipocyte-specific genes and accumulation of lipid droplets in hypoxic BMSCs suggest that, at least in vitro, the adipogenic program of BMSCs requires a rather hypoxic microenvironment and that prevention of BMSC differentiation in their physiologic niche requires the activity of specific regulators upstream of HIF-1α [41,42]. One of the candidate regulators is the mTOR pathway that mediates maintenance of the undifferentiated state via parallel intracellular signaling systems including the HIF-1α pathway [46]. Interestingly, one of the activating stimuli of the mTOR complex 1, an entity of mTOR, is glutamate of which elevated intracellular levels due to its hypoxia-affected metabolism can also mediate mTOR activation under hypoxia [47,48]. In bone marrow hemopoietic cells, activated mTOR induces the expression of Interleukin-6 (IL-6), the same cytokine that is most abundantly expressed in BMSC and that inhibits their adipogenic and chondrogenic differentiation [46,49]. Interestingly, IL-6 and the membrane-bound IL-6 receptor (IL-6R) are both induced during osteogenic differentiation of BMSCs, suggesting not only the role of hypoxia/mTOR/IL-6 axis in the regulation of BMSCs stemness but the comprehensive effects of oxygen on the signaling between BMSC and their neighboring bone marrow-resident cells as well [50]. Surprisingly, in-depth analysis of the BMSC secretome confirmed a conserved pattern of only a handful BMSC-secreted cytokines raising the existence of alternative BMSCs-employed signaling mechanisms potentially affected by hypoxia within the bone marrow [49]. Indeed, data indicate that BMSC maintain extensive cell-to-cell communication by shedding large quantities of extracellular vesicle that received particular attention recently due to their possible therapeutic importance [49,51,52].

### 1.4. Extracellular Vesicles

**Figure 3:**
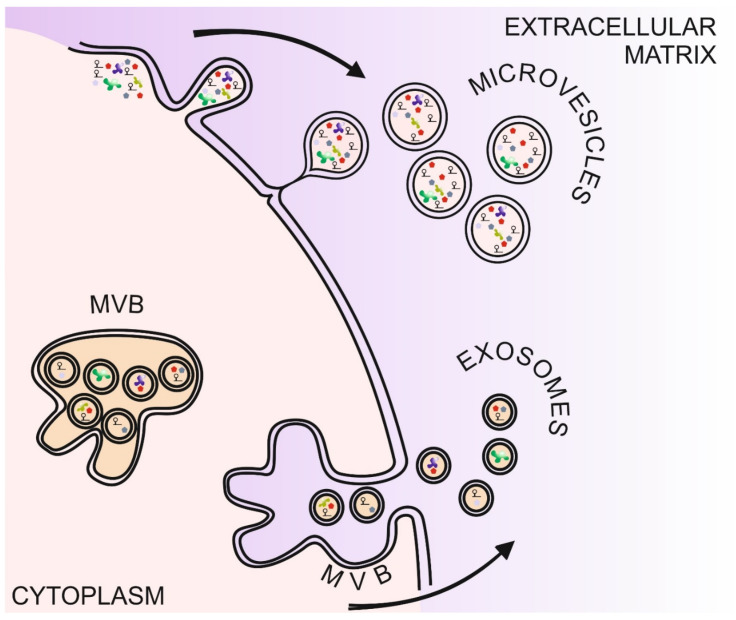
Schematic overview of the genesis of extracellular vesicles. The two forms of extracellular vesicles are generated via different pathways. Microvesicles are formed by budding of the cell membrane whereas exosomes are produced inside of the multivesicular bodies. These, then, can fuse with the cell membrane, releasing the exosomes into the extracellular milieu. Abbreviations used are: MVB: multivesicular bodies.

The term extracellular vesicles (EV) refers to membrane-bound circular organelles with various sizes in the nanometer range released by, apparently, every mammalian cell type [53] (Figure 3). Secreted EVs can fuse with the plasma membrane of various target cells, provoking a wide range of biological responses representing a higher-level complexity of cell-cell interactions. They include the exosomes that range between 40 and 100 nm in diameter, similar to that of the vesicles generated within the multivesicular bodies (MVBs) [54]. Indeed, data indicate that exosomes are formed within MVBs by inward budding and released to the extracellular matrix following the fusion of MVBs with the plasma membrane [55]. In contrast to the plasma membrane, however, exosomal membranes show differential lipid composition [56]. They are enriched in cholesterol, sphingomyelin and hexosylceramides while phosphatidyl-choline and -ethanolamine are less abundant in exosomal membranes [57,58,59]. Proteins are also regularly detected in exosomes but their protein composition seems to be more heterogenous depending on the source of the vesicles and the analytic methods applied [60]. Still, the plasma membrane microdomain-clustering tetraspanins, the endosome biogenesis-related annexins, heat shock proteins and the lipid raft-related flotillin seem to be stably enriched components of the exosomes [57,61,62,63]. Data suggest that the differential tetraspanin composition of exosome membranes is one of the determinants of the target-cell specificity of the exosomes [64]. Besides their common membrane-related protein elements, a wide range of intracellular proteins have also been assigned to exosomes including cytoskeleton elements [65,66], metabolic enzymes [67,68] or canonical signaling molecules [69]. Ribonucleic acids including messenger RNAs and various non-coding RNA transcripts have also been reported to be present in exosomes [64,70,71,72,73,74]. The physiologic importance of the RNA content of exosomes is highlighted by the findings that exosome-encapsuled RNA species seem not only to be selectively incorporated into the exosomes but could also be translated in recipient cells [70,75]. Current experimental data suggest that the composition of the EV cargo is determined by both the type and the microenvironment of the parental cell [76].

Besides exosomes, mammalian cells also release larger EVs, termed microvesicles (MV), that are typically up to 1 um in diameter [55]. Besides their size difference compared to exosomes, another defining hallmark of MVs is that their biogenesis is linked to the outward budding of the plasma membrane [77,78,79] (Figure 3). Formation of MVs seems to be under the control of intricate mechanisms that include differential membrane lipid compositions and rearrangement of the microfilament system alike [80,81]. The latter one is mediated by a number of pathways including the small GTPase ARF6/Phospholipase D/ERK/Myosin light chain kinase pathway that mediates phosphorylation of the Myosin light chain resulting in MV release in cancer cell models [82]. Data also suggest that additional microfilament regulatory pathways, such as the RhoA/LIM kinase/cofilin pathway are also involved in MV formation [83,84]. The apparently central role of the membrane-bound small GTPases in the regulation of MV biogenesis suggests that extracellular stimuli can directly influence MV formation. Indeed, hypoxia induces MV shedding in a HIF1-dependent manner via induction of the small GTPase *RAB22A* [85]. Although the underlying molecular mechanism might be slightly different depending on the cell type, the hypoxia-responsive nature seems to be a common feature of EV biogenesis [85,86,87,88].

### 1.5. BMSC-Released Extracellular Vesicles

Similar to other mammalian cell types, hBMSCs also secrete various EVs enriched in proteins, metabolites and RNA species [89,90,91,92]. Proteomics profiling revealed that the BMSC-secreted EVs both carry canonical exosome markers, such as the tetraspanins, and BMSC-specific cargo [93]. These include BMSC markers, ion- or protein-transporters, transcriptional- and cell-cycle regulators, angiogenic factors and proteins associated with the Wnt signaling pathway or the organization of the extracellular matrix alike [94]. Among the RNAs, coding and non-coding species have also been reported in BMSCs-derived EVs, suggesting their complex effects on target cells. Indeed, BMSC-derived EVs have been reported to promote the paracrine transfer of both collagen type VII collagen (COL7A1) and its functional mRNA to support secretion and de novo synthesis of collagen type VII in COL7A1-deficient neighboring fibroblasts [95]. In contrast, BMSC-released EVs can reduce the expression of collagen I or the transforming growth factor-1β, critical elements of the fibrotic response of connective tissue [96]. Moreover, the fibroblast function-modulating effects of BMSC-derived EVs include the influence of processes such as the ossification of ligaments via their miRNA content, confirming the multifunctional nature of the BMSC-produced EV population [97]. Current data suggest that there are multiple mechanisms behind the differential effects of BMSC-derived EVs on fibroblast. On one hand, cargo composition of BMSC EVs is influenced by the extracellular milieu of the EV-releasing cell, as was seen in osteogenically-induced BMSCs that secrete EVs that promote osteogenic engagement of naïve BMSCs via their non-coding RNA cargo such as miR-29b-3p and miR-22-3p [76,98,99]. On the other, data also indicate that BMSCs release functionally unequal EV populations that allows differential effects in a given EV cohort [100].

Based on the complex cargo composition of BMSC EVs, one can speculate that the spectrum of their target cells is not necessarily limited to fibroblast. Indeed, BMSC-derived EVs seem to contribute to the maintenance of the hemopoietic stem cell population, one of the central functions assigned to BMSCs residing in their physiologic niche [101,102]. In addition, BMSC EVs seem to mediate similar anti-inflammatory properties than that of their parent cells [103]. These effects, however, are mediated, at least partly, by different miRNA populations including the miRNA-21 and miRNA-34a that target *KLF4* and the cyclin I/ATM/ATR/p53 axis, respectively [104,105]. Besides their anti-inflammatory effects in chronic inflammatory conditions, BMSC-derived EVs also display similar activities in graft versus host models by influencing the ratio of T cell populations biasing regulatory over cytotoxic species [106]. Moreover, observations that BMSC EVs show non-coding RNA-mediated pro-survival effects on antibody secreting cells as well as promote M2 macrophage polarization in chronic inflammatory models underpin the idea that they can target a wide range of cells independently of their histological origin [107,108,109].

The non-coding RNA content of BMSC EVs includes miRNA that further widens the range of the potential target cells by influencing neuroinflammatory responses [110]. These include the miRNA-183-5p and microRNA-221-3p that both have been assigned to the neuroprotective effects of BMSC EVs [111,112]. Their protective effects in ischemia/reperfusion injury are also not restricted to neuronal tissues as has been reported in the hepatocellular context but, apparently, are mediated by another non-coding RNA cargo, the miRNA-146a-5p [113,114]. Moreover, in mouse models, BMSC EVs induce *VEGFR1* and *-2* expression of endothelial cells and promote endothelial tube formation in vitro [115]. These EVs were found to be enriched with the VEGF and miR-210-3p, suggesting that the pro-angiogenic effects on endothelial cells are mediated by concentrated factors that are capable of triggering the pro-angiogenic program of target cells at multiple levels [115]. The positive effects of BMSC EVs on hindlimb ischemia in in vivo models raise the question if the intimate relationship between BMSC and hypoxia is reflected in the physiologic potential of BMSC-released EVs.

### 1.6. Hypoxia and the BMSC-Released Extracellular Vesicles

Considering that hypoxia has widespread and profound effects on BMSCs and environmental stimuli fundamentally affect the cellular secretome, it is not surprising that oxygen depletion significantly affects the composition of BMSC EVs. The primary candidates to mediate this effect are, obviously, the HIFs. Indeed, HIF-1α directly induces, for instance, miR-210, a known BMSC EV cargo that secreted in elevated amounts in EVs released by hypoxic BMSCs [116,117,118]. In accordance, miRNA-210 exert pro-angiogenic effects via induction of *VEGF* in endothelial cells, providing a potential explanation of the superior effects of EVs released by hypoxic BMSCs observed in ischemia/reperfusion injury models [119,120,121,122]. Besides miRNA-210, there is a growing number of evidence of further microRNAs that may play a role in the BMSC EVs mediated protection upon ischemia/reperfusion. These include miRNA-22 that is also enriched in EVs of hypoxia-exposed BMSCs and directly transferred to cardiomyocytes via an EV-mediated manner [123]. Following its uptake, miRNA-22 downregulates the epigenic regulator MECP2 and contributes to the reduction in the post-ischemic fibrotic response of the myocardium [123,124]. Another microRNA, miRNA-26a, also seems to contribute to cardioprotection upon ischemia/reperfusion by targeting the glycogen synthase kinase 3beta (GSK3β) encoding *GSK3B* [125]. Moreover, in rodent models, miRNA-149 and Let-7c-5p were reported to be enriched in EVs of hypoxia-preconditioned BMSCs where, following their horizontal transfer to cardiomyoblasts, they repress FAS ligand expression, rescuing them from hypoxia/reoxygenation-induced apoptosis [126]. Finally, miR-125b, another miRNA species enriched in EVs secreted by hypoxia-preconditioned BMSC, efficiently repress the pro-apoptotic *TP53* and *BAK1* genes in ischemia/reperfusion models, clearly indicating that the cardiomyocyte-protective effects of hypoxic BMSC-released EVs are mediated by multiple, simultaneously influenced pathways [127].

EVs of hypoxia-preconditioned BMSCs were found similarly advantageous in neurodegenerative disease models over EVs produced by normoxic BMSCs but the underlying mechanisms are also yet to be elucidated [128]. One of the potential candidates to mediate this effect is miRNA-21 that is induced in hypoxic BMSC and mimics anti-inflammatory effects of the hypoxic BMSC EVs in the recipient neuronal cells [129]. In response, miRNA-21 levels are increased in the target cells as well, but whether the excess amounts of miRNA-21 species are directly transferred from hypoxic BMSCs via EVs or other cargo induces miRNA-21 expression in the recipient cells is not clear [129]. Data also suggest that the neuroprotective effects of hypoxic BMSC-released EVs are not exclusively mediated by intraneuronal mechanisms. Instead, EVs excreted by hypoxic preconditioned BMSCs exert complex, intra- and supracellular effects, simultaneously affecting neuronal signaling pathways and activation of the astrocytes and microglia [129]. Moreover, data indicate that the anti-inflammatory nature of EVs derived from hypoxia-preconditioned BMSCs is not restricted to immunocompetent cells of the central nervous system. Indeed, they were also found to efficiently reduce the number of infiltrating white blood cells upon endotoxin-induced acute lung injury and, for this effect, a brief exposure of BMSCs to hypoxia is already sufficient [130].

Although these data assign potential therapeutic importance to hypoxic BMSC-secreted EVs, some recent experimental data suggest that their versatile nature might be disadvantageous in the cancer context. Indeed, various cargo of BMSC EVs have been reported as potential mediators of lung cancer progression. These include miRNA-328-3p, miRNA-193a-3p, miRNA-210-3p and miRNA-5100 and miR-21-5p that were all found to contribute to cancer cell growth and mobility hijacking various intracellular pathways of the recipient cancer cells [118,122,131] (Table 1).

## 2. Discussion

Extracellular vesicles excreted by BMSCs have received growing attention over the past few years. These include studies that focused on the composition of EVs released BMSCs exposed to low oxygen conditions that found these “hypoxic” EVs superior to their “normoxic” counterparts, raising the question if the beneficial effects of “hypoxic” EVs are due to the hypoxic intracellular composition of the parent cells. This is a fascinating question suggesting that understanding the composition of “hypoxic” vs. “normoxic” EVs could provide valuable information on the key regulators of pathologic states. Indeed, identification of microRNA-148a-3p as the mediator of the beneficial effect of BMSC EVs on osteonecrosis confirmed the role of SMURF1 in the pathogenesis [132]. Similarly, identification of targets of the BMSC EV cargo miRNA-375, that mediate anti-cancer effect on cervical cancer, might reveal further components in the transformation of cervical epithelium [92].

One could speculate that analysis of the BMSC EV composition might shed light on BMSC physiology as well. Indeed, miRNA-15b, that is enriched in BMSC EVs revealed intracellular regulators of osteogenic differentiation confirming that BMSC EVs could provide invaluable information on BMSC-related processes [133].

Interestingly, most of the current data on the composition of “hypoxic” BMSC EVs focus on their microRNA cargo and we have no data on the effects of their protein or metabolite content on the recipient cells. In addition, considering the fundamental intracellular changes in hypoxic BMSCs, one can speculate that the protein and metabolite composition of hypoxic EVs reflect similarly profound alterations. Thus, it would be interesting to see if cargo proteins of the “hypoxic” EVs contribute to the hypoxic adaptation of recipient cells. Similarly, the fundamental role of metabolites such as succinate or the α-ketoglutarate in the regulation of the HIF pathway raises the question if these metabolites are present in “hypoxic” BMSC-secreted EVs in differential concentrations and if they influence the HIF pathway of recipient cells.

The beneficial effects of BMSC-derived EVs on various human pathologies naturally fuel the idea of their future use in clinical applications. For the successful implementation of BMSC EV-based cell-free modalities in the clinical practice, however, therapeutic EVs need to be standardized. Since the molecular signature and physicochemical properties of EVs reflect the same of the parental cells, one of the candidate solutions for the standardization problem seems to be the production of off-shelf EV-based therapeutic agents instead of the use of flask-cultivated autologous BMSC-derived ones [134]. Implementation of hollow-fiber bioreactors for standardized and upscaled production of BMSC-derived EVs is one of the promising steps toward EV-based off-shelf products [135]. To bias the potential of the manufactured EVs toward the desired therapeutic effect, however, more detailed mapping of the cargo composition of EVs under differential culture conditions seems to be inevitable. Understanding the payload constellation of the hypoxic BMSC EVs, with particular attention to the dissection of their cargo exerting anti-ischemic and anti-inflammatory effects from the those with oncogenic ones, seems to be critical to support both current and future efforts to produce off-shelf nanovesicles that efficiently but safely mirror the superior therapeutic effects of hypoxic BMSC EVs.

## Figures and Tables

**Table 1:** Summary of the cargo molecules enriched in EVs released from hypoxic BMSCs.

Hypoxic EV Payload	Observed Effects	References
miR-210	induces expression of VEGF resulting in angiogenesis	[116,117,119,120,121,122]
miRNA-22	downregulates MECP2 contributing to the reduction in the post-ischemic fibrotic response	[123,124]
miRNA-26a	targets the glycogen synthase kinase 3beta (GSK3β) encoding GSK3B	[125]
miRNA-149	represses FAS ligand expression rescuing them from hypoxia/reoxygenation-induced apoptosis	[126]
Let-7c-5p	represses FAS ligand expression rescuing them from hypoxia/reoxygenation-induced apoptosis	
miR-125b	represses the pro-apoptotic TP53 and BAK1 further indicating protective effects	[127]
miR-21	affects neuronal signaling pathways and activation of the astrocytes and microglia	[129]
miRNA-328-3p, miRNA-193a-3p, miRNA-210-3p miRNA-5100 miR-21-5p	contribute to cancer cell growth and mobility	[118,122,131]
